# Research on Simulation Optimization of MEMS Microfluidic Structures at the Microscale

**DOI:** 10.3390/mi16060695

**Published:** 2025-06-11

**Authors:** Changhu Wang, Weiyun Meng

**Affiliations:** 1 School of Physics and Physical Engineering, Qufu Normal University, Jining 272000, China; z2013168@sdaeu.edu.cn; 2 College of Mechanical and Electronic Engineering, Shandong Agriculture and Engineering University, Jinan 250100, China

**Keywords:** microfluidic system, finite element simulation, Micro-Electro-Mechanical System, micropump, structural optimization

## Abstract

Microfluidic systems have become a hot topic in Micro-Electro-Mechanical System (MEMS) research, with micropumps serving as a key element due to their role in determining structural and flow dynamics within these systems. This study aims to analyze the influence of different structural obstacles within microfluidics on micropump efficiency and offer guidance for improving microfluidic system designs. In this context, a MEMS-based micropump valve structure was developed, and simulations were conducted to examine the effects of the valve on microfluidic oscillations. The research explored various configurations, including valve positions and quantities, yielding valuable insights for optimizing microfluidic transport mechanisms at the microscale.

## 1. Introduction

Microfluidic systems enable the precise control and manipulation of droplets and flow on a microscale [1,2], facilitating separation and movement through finely tuned liquid flow [3]. By integrating principles from micro-mechanics, fluid dynamics, and smart materials, these systems stand out for their compact design and precise fluid handling capabilities [4,5]. Applications span from lab-on-a-chip technologies and microbiology to cooling systems in high-performance electronics and targeted drug delivery. Micropumps are essential for fluid delivery in microfluidics [6,7], supporting diverse uses such as biological fluid transport and cooling in miniaturized electronic devices.

Microchannels are the main components of microsystems. When multiphase liquid flows in laminar flow in microchannels, mixing can occur between adjacent phases through diffusion [8]. They have been widely used in microchemical systems with complex multiphase flows, such as in medical [9] and pharmaceutical processes [10,11,12]. Microfluidic systems have a wide range of applications in sealing, non-contact sensing, and other fields [13,14,15,16,17]. By changing the inlet flow velocity of multiphase fluids in microchannels, various interfacial systems such as laminar flow and annular flow can be formed within the channel. Two-phase flow separation in microchannels is influenced by interactions such as inertial forces, viscous forces, and surface tension [18,19], while fluid velocity, viscosity, cross-sectional shape, and microchannel geometry affect fluid flow patterns and mass transfer rates [20,21]. By controlling the contact time of the solution near the interface, the stable changes of the interface can be controlled. When two fluids come into contact, they will mix and eventually produce a uniform fluid. The existing research has mostly focused on numerical simulations of interface stability in microchannels and the influence of two-phase fluid convection and diffusion on interface width, lacking mechanical models and specific mechanical analyses such as thermodynamics. However, it has laid the foundation for the interface dynamics and thermodynamics related to complex fluids in microfluidics.

## 2. Working Principle of Microfluidic Systems

Microfluidic systems are systems used to control and manipulate small fluids, and their working principles mainly include the following aspects. (1) Microfluidic systems manipulate the flow of fluids through microchannels, which are typically in the micrometer range and precisely manipulate fluids. (2) Generally, pressure drive or electric potential drive are used to regulate fluids. Pressure drive applies pressure through micro pumps or pneumatic systems to make fluids flow in microchannels, while electric potential drive controls fluid motion through electric field effects. (3) Separate fluids through the branching structure of microchannels and special surface treatment techniques. By adjusting the shape of the flow channel or applying a specific electric field to separate different components, microfluidic systems use micro stirrers or micro chaotic structures to promote rapid and uniform mixing of fluids. Microfluidic systems are typically equipped with microsensors to measure parameters such as temperature and pressure of fluids, enabling real-time monitoring and control of fluids [22,23,24]. Microfluidic systems are widely used in biological analysis, chemical reactions, drug screening, and other fields [25,26]. The advantage of these systems lies in their use for processing extremely small amounts of samples, as well as high flow accuracy and analysis efficiency.

### 2.1. Working Principle and Features of Micropumps

Micropumps regulate fluid outflow using diffusion and contraction tubes that create varying fluid dynamics due to differences in their capacities and structures. These variations produce distinct inflow and outflow rates, resulting in differential fluid flow [27,28]. The pumps operate by employing moving components to induce periodic vibrations. Each cycle consists of two modes: “suction” and “pumping”. During the suction mode, the pump chamber expands upward under external force, increasing its volume and lowering internal pressure relative to the outside. At this stage, the inlet acts as a diffusion outlet [29,30] and the outlet functions as a contraction tube, causing the fluid flow rate in the diffusion tube to exceed that in the contraction tube, thereby enhancing the inflow. In the pumping mode [31,32], the chamber contracts downward, reducing its volume and raising the internal pressure. At this point, the inlet functions like a contraction tube and the outlet behaves as a diffusion tube, allowing fluid to exit the chamber and complete the transport process.

The pump chamber contains oscillating diaphragms on either side that adjust the chamber volume to draw in and expel fluid [33,34,35]. Through their oscillation, these diaphragms modulate the chamber volume, effectively forming a one-way valve with the combined action of the diffusion and contraction tubes. This micropump design, utilizing diffusion and contraction tubes, unifies the driving source, transmission part [36,37], and pump body traditionally found as separate elements in conventional pumps. However, moving components may incur losses from pressure and are susceptible to wear, fatigue damage, and specific challenges when handling certain fluids [38,39]. At the microscale, advancements in driving technology for micropumps continue to address these evolving demands.

### 2.2. Applications for Micropumps

Micropumps are highly effective at operating under high frequencies, making them indispensable for transporting liquids with suspended particles or biological materials. These pumps hold considerable promise for applications in chemistry [40], pharmaceuticals, and biomedicine. In microfluidic channels, slight disruptions from rough wall surfaces can influence fluid dynamics within the system, leading to vortex structures in the near-wall regions of the flow fields. The fluid flow typically remains in a laminar state, where internal friction shear stress follows Newton’s law of internal friction. Key parameters of the microfluidic system, such as velocity distribution [41], flow rates, and energy losses during laminar flow, can be determined using mathematical methods.

## 3. Oscillatory Fluid Motion

In this study, the “Fluid-Solid Coupling” interface within COMSOL 6.1 is employed to analyze fluid flow and the resulting deformations within valve structures. The “Global Ordinary Differential and Differential Algebraic Equations” module facilitates time-based numerical calculations, tracking fluid flow throughout each phase of the pumping cycle across the microfluidic system. The reciprocating pumping mechanism induces oscillatory fluid motion, transforming this oscillation into a directed net flow. Within the system, the oscillatory pumping action is achieved through a piezo-oscillator that vibrates the membrane, periodically adjusting the volume of the microchamber.

### 3.1. Microscale Model Geometry

The model geometry described in this paper comprises a horizontal channel and a vertical chamber. The horizontal channel spans 1000 μm in length and 100 μm in height, with a vertically oriented chamber connected at its midpoint. Within the channel, two angled valve flaps are centrally positioned, creating a narrow, upright structure. These valve flaps, influenced by oscillatory fluid motion, generate a net fluid flow from left to right through the channel. As fluid moves from left to right, it enters the narrow section in the upper half of the channel after passing through the valve flaps. This fluid movement applies viscous resistance and pressure against the internal channel structures, resulting in a net force on both sides of the internal configuration. Made from a flexible material, the valve flaps deform under external forces, changing the direction of fluid flow. The numerical results of fluid flow need to be calculated based on known conditions.

Table 1 shows the microchannel model configuration and obstacle data. At the bottom of the channel, two angled valve flaps partially obstruct the fluid flow along the channel’s length. These valve flaps are separated by a specific distance, with the midpoint of the space between them aligned with the midpoint of the channel. Positioned at a 60-degree angle from the horizontal plane, each flap measures 6 μm in width and 56 μm in height, featuring a semicircular top edge. They are located 200 μm and 700 μm from the left boundary of the channel, respectively. The fluid within the channel is modeled as a water-like substance with a density of 1000 kg/m^3^ and a dynamic viscosity of 0.001 Pa·s.

### 3.2. Flow Process Analysis

The numerical simulations in this study are conducted using the “Fluid-Solid Coupling” multiphysics interface. The inlet boundary is positioned above the vertical chamber, where the inlet flow velocity is input with a sinusoidal variation with a period of 2 s. The left and right boundaries of the channel act as the outlet boundaries. At each outlet, the flow rate is calculated by integrating the horizontal fluid velocity component (the dependent variable) and then multiplying it by a surface length scale of 10 μm. This approach allows for determining the flow rate difference between the left and right outlets, with a positive net flow value indicating a left-to-right movement. By analyzing this data, this study calculates the net flow variation over time, ultimately providing the total volume flow of the pump in one cycle. This analysis specifically examines the transient dynamics throughout a cycle, with a focus on the two complete oscillations of the inlet velocity.

According to Poiseuille’s law, the flow resistance of a fluid channel in a laminar state is the ratio of pressure drop Δ*P* to volumetric flow rate *Q*. This can be mathematically expressed in the form below:

(1) Rc=ΔPQ=ΔPAU

Abrupt changes in flow can arise due to the deformation of the valve flap and the corresponding fluid feedback. To manage this, the solver employs larger time steps when the solution remains relatively stable. In the fluid–solid coupling model, sudden changes in fluid flow may occur, so setting a maximum time step aids in accurately capturing these rapid variations.

The valve disc is mainly subjected to the combined effects of fluid hydraulic force, water impulse force, and friction force. As shown in the figure, the region with high fluid velocity is located at the gap above the valve disc. The maximum flow velocity increases linearly with the inlet pressure and is ejected forward in the form of a jet. The back of the valve disc is surrounded by liquid, and there is no instant fluid filling, forming a local vacuum. However, as the valve disc continues to rotate and open, the compressed vacuum area gradually decreases until the fluid completely fills the vacuum area. The pressure at the front end of the valve disc is significantly higher than that at the back end and, due to the asymmetric structure of the valve body, the pressure distribution in the flow field is uneven, which can easily cause vibration. The maximum pressure distribution area of the valve disc is located above the valve disc due to fluid impact, then the impact force on the valve disc in the axial direction decreases, resulting in a relative decrease in the velocity of the valve disc movement. Subsequently, due to a further increase in inlet pressure, the velocity of the valve disc movement shows an increasing trend. The height of the channel is slightly larger than the length of the valve disc. This project mainly explores the influence of valve disc structure on fluid flow. Under the impact load of liquid, the valve disc will produce a stress accumulation phenomenon at the front end of the valve disc, which will have a significant impact on the strength of the valve disc. The temperature rise flow field in the channel will also be greatly affected. The impact load of fluid on the sealing surface under pressure is relatively large.

**Figure 1:**
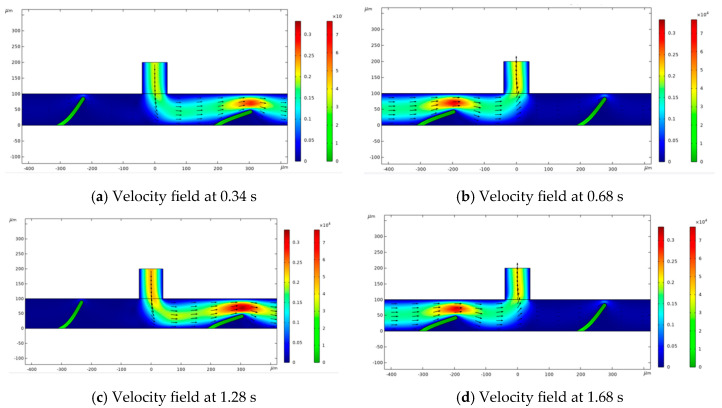
Flow vectors superimposed on velocity contours at different times.

Figure 1 illustrates changes in flow velocity at four distinct time points: t = 0.34 s, 0.68 s, 1.28 s, and 1.68 s. The streamlines show the fluid flow positions, with the color gradient from blue to red indicating an increase in flow velocity from lower to higher levels. As fluid moves through the channel, the valve flap boundary shifts vertically and horizontally, with the highest fluid velocity occurring near the flap. The degree of flap deformation correlates with the inflow velocity; as the flow rate reduces, the force exerted by the fluid weakens, resulting in less deformation. The fluid enters from the left side along the horizontal channel and passes through the constricted area above the flap, which bends under the influence of fluid pressure and viscous resistance. This study explores variations in flow patterns in response to the bending and deformation of the flap.

(2) $$\frac{\Delta p}{l}=f_{D}\times \left(\frac{\rho }{2}\right)\times \left(\frac{U^{2}}{D}\right)$$

where Δ*p* is the pressure drop, *l* represents the length, *ρ* is the fluid density, *U* is the average fluid velocity, *D* denotes the hydraulic diameter of the fluid channel, and *f_D_* is the Darcy friction factor. In the laminar state, this factor is defined as follows:

(3) $$f_{D}=\frac{64}{R_{e}}=\frac{64\nu }{DU}$$

where *Re* is the Reynolds number and *ν* denotes the dynamic viscosity.

As fluid enters through the inlet along the horizontal channel, both valve flaps simultaneously bend toward the channel’s bottom. However, their bending amplitudes differ, creating a condition that allows fluid to flow more readily toward the right outlet.

### 3.3. Analysis of Flow Process at Different Positions of the Valve Flaps

As the fluid flows upward, it moves from the horizontal channel into the vertical chamber, causing the two valve flaps to bend outward in opposite directions. During this upward movement, fluid flows from the channel toward the inlet, with each valve flap bending differently. The right valve flap, having a greater bending amplitude, restricts flow more effectively than the left valve flap, allowing fluid from the left to enter the vertical chamber and move from left to right.

In calculating the net flow rate within the channel, left-to-right flow is assigned a positive value, while right-to-left flow is assigned a negative value.

**Figure 2:**
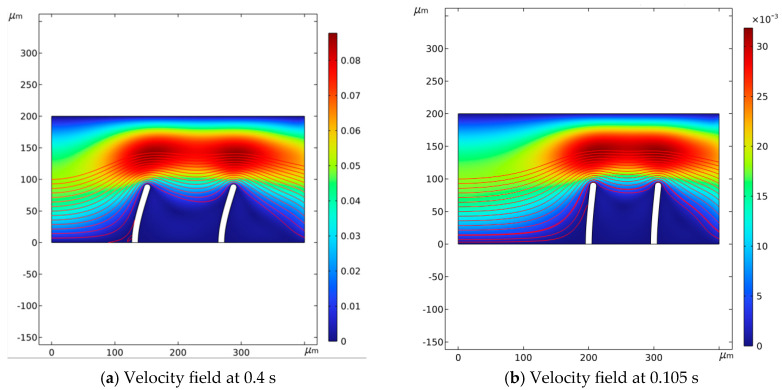
Velocity field when the valve flaps are at different positions.

Figure 2 illustrates the fluid flow velocity and velocity field at two specific moments: 0.105 s and 0.4 s. Streamlines indicate the flow direction, while color variations represent different flow velocities. As fluid moves, the valve flaps act as dynamic boundaries; fluid velocity peaks near the flaps, causing the greatest variation in fluid behavior. Flow velocity impacts fluid deformation; as flow velocity declines, deformation decreases. By the end of a cycle, the inflow and structural deformation settle into steady-state values and the flow velocity reduces to zero.

**Figure 3:**
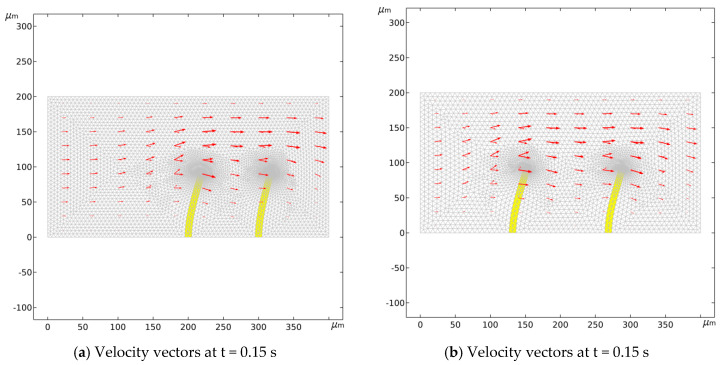
Velocity vectors when the valve flaps are at different positions.

Figure 3 illustrates the conditions at time t = 0.15 s. Comparing the grid velocity and valve flap deformation at various positions reveals the direction and changes in the velocity field post-deformation, with clear horizontal variations in fluid movement. On the left side of the valve flap, grid elements are stretched, resulting in an unclear velocity field. Conversely, on the right side, the grid elements compress horizontally, forming a distinct fluid velocity field and demonstrating significant velocity changes as fluid flows past the valve flaps. The positioning of the valve flaps notably influences the velocity field’s variation. Flaps placed further to the left lead to greater fluctuations in the fluid velocity field.

**Figure 4:**
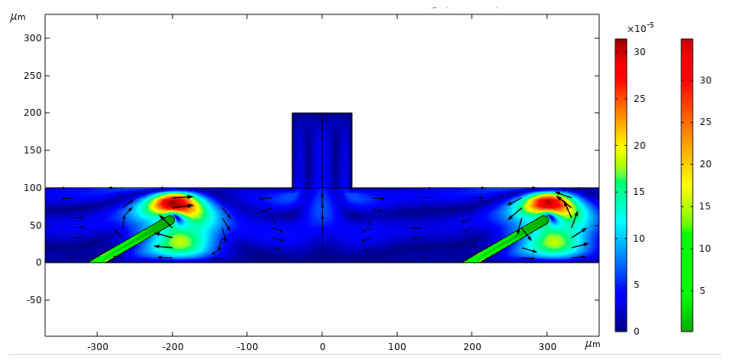
Seepage stress contour.

Figure 4 displays the stress distribution in the system at t = 2 s (as the system nears its steady state). The seepage stress streamlines form a radial pattern, with peak stress values appearing at the top of the valve flap and gradually diminishing downward, followed by a slight increase near the bottom. This shows that stress levels are significantly higher around the valve flap than in other regions. Throughout fluid seepage, the flow direction continually changes, with the fluid moving from areas of high to low pressure. In accordance with Darcy’s law, the flow velocity is directly proportional to the pressure gradient, meaning that as the pressure drop rises, so does the flow velocity.

The fluid maintains a laminar state due to the small size of the channel and the low Reynolds number. Vortices develop in the confined area behind the valve flap, with their size and location dictated by the flow velocity, while the degree of deformation is influenced by the inflow velocity. There is a net fluid flow from left to right, although this flow is obstructed by the valve flap.

Pressure variations exhibit both oscillating and stationary states at identical pressure levels, transitioning from low to high and back to low. During periods of high pressure, a lag phenomenon is observed. Under dynamic conditions, the liquid collides with the surfaces of the valve flaps, transforming kinetic energy into elastic potential energy during the impact, alongside associated energy losses.

**Figure 5:**
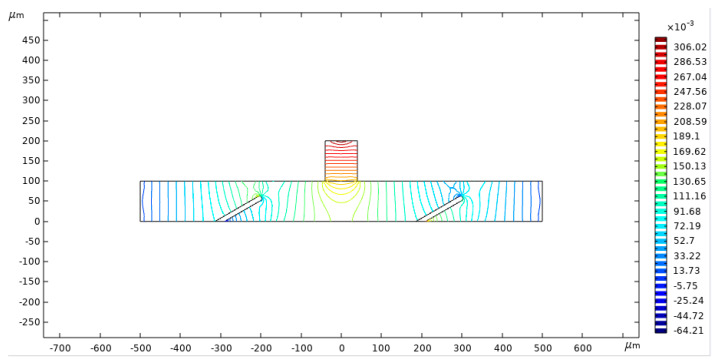
Pressure contour of the fluid flow.

The continuity equation of a micro pump is as follows. The difference between the inlet flow and outlet flow of the micropump is solved by differential equations. The volume of the pump cavity is derived with respect to time, and the fluid density is derived with respect to time. Finally, the following micropump continuity equation is sorted out.

(4) $$q_{i}-q_{0}=\frac{d_{\nu c}}{d_{t}}+\frac{\nu_{c}}{\rho }\frac{d_{p}}{d_{t}}=\frac{d_{\nu c}}{d_{t}}+\frac{\nu_{c}}{k_{eff}}\frac{d_{pc}}{d_{t}}$$

where $q_{i}$ is the inlet flow rate of the micropump;

$q_{0}$ is the outlet flow rate of the micropump;

$v_{c}$ is the volume of the pump cavity;

ρ is the fluid density;

$K_{eff}$ is the effective bulk modulus of the fluid;

Figure 5 presents the pressure contour of fluid flow at t = 2 s. The streamlines illustrate the variation in flow pressure, while the color gradient indicates the magnitude of the flow pressure. The highest flow pressure is observed along the central vertical axis, gradually decreasing as it extends downward. Near the outlet, positioned outside the valve flap, the flow pressure shows a gradual decrease followed by an increase in the opposite direction.

### 3.4. Experiment

The experiment measured the pressure drop along the flow path of the fluid in microchannels with different cross-sectional shapes and sizes and studied the influence of flow resistance under different cross-sectional shapes of microchannels.

Table 2 shows the cross-sectional shape and data for the microchannels. The experiment compared the frictional resistance and Reynolds number variation of microchannels with three different cross-sectional shapes: rectangular, semi-circular, and triangular. When the cross section was rectangular, the critical Reynolds number for channel 1 to undergo transition was found to be 800, and its friction coefficient in the laminar flow region was basically consistent with the conventional theoretical value. Channel 2 exhibited flow transition at a Reynolds number of around 600. When the Reynolds number of channels 1 and 2 was greater than 2000, the flow transformed into fully developed turbulence. The resistance coefficients of the two channels were basically the same in the laminar and turbulent flow regions, but the numerical values were slightly different. When the cross section is semi-circular, the transition point Reynolds number of channel 3 is around 1000 and the transition phenomenon is more pronounced than that in channel 4. In the laminar flow region, the friction coefficient is smaller than the conventional theoretical value and channel 4 exhibits a transition phenomenon at a Reynolds number of around 700. The flow in both channels transitions to fully developed turbulence at a Reynolds number of 2000. When the cross section is triangular, the transition point between channel 5 and channel 6 occurs around 900. When the cross-sectional shape of the microchannel is different, the resistance coefficient is also different. When the cross-sectional shape is the same, the resistance coefficient decreases with the decrease in equivalent diameter.

**Figure 6:**
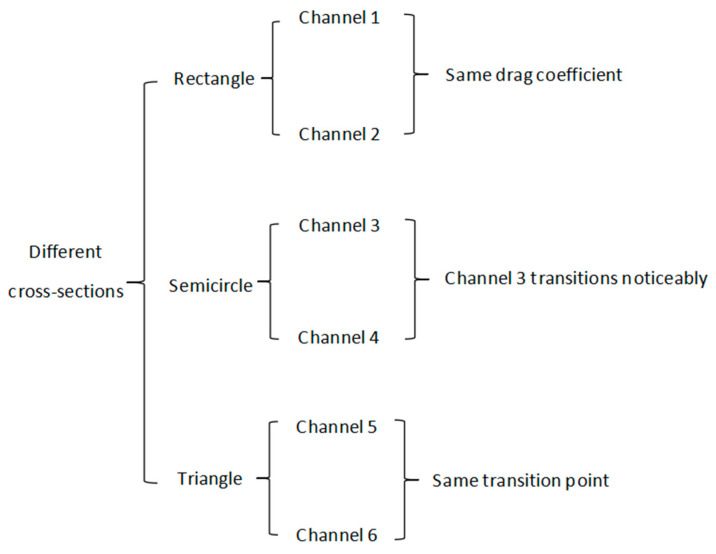
Flow process diagrams of different cross sections.

Figure 6 shows the flow of fluid through channels of different cross-sectional shapes and widths. The microchannels used in this experiment were fabricated using a 3D printing system, which can basically meet the machining accuracy requirements of the microchannels in this experiment. The 3D printing material adopts photosensitive resin material to meet the precision requirements of microchannels in this experiment. The production of microchannels mainly includes organizing various pieces of microchannel data for 3D printer preparation work. After determining the general structure and characteristics of the microchannel model and then using a 3D printing system for production, this study mainly explores the fluid distribution in different cross sections, such as triangles and rectangles. By studying the width, length, height, shape, and structure of the microchannel, the resistance and friction of the microchannel under the influence of each cross section are clarified. After fixing the number of microchannels processed by the printer, the fluid is injected into the microchannels at a stable flow rate; finally, the liquid is recovered through a recovery tube. In addition, we ensure the dryness of the microstructure before the experiment. When conducting multiple experiments using the same channel, we discharge the liquid after each experiment to keep the microchannel dry. The critical Reynolds number for flow transition in this article is between 700 and 1000. When the flow is fully turbulent, the drag coefficient of triangular and semi-circular interface microchannels is basically equal but smaller than the frictional drag coefficient of rectangular cross section channels.

## 4. Conclusions

Modern science has evolved to encompass research across a range of scales, including macroscopic, microscopic, and nanoscale dimensions, along with multiple temporal and spatial variables. The advancement of multiscale research significantly benefits everyday life. Computer simulations have gained immense importance in scientific investigations, particularly within the medical field, encompassing areas such as physiology, pathophysiology, and surgical treatment. This paper explores the application of computational fluid dynamics in microfluidic systems and examines the influence of obstacles, such as valve flaps, on fluid flow at the microscale. The study reveals that when oscillatory flow is injected into channels with flexible valve flaps, the flaps deform in response to fluid impact, consequently altering the flow direction. Utilizing a “Fluid-Solid Coupling” multiphysics interface, the research delves into the fluid dynamics characteristics of both the fluid and the valve flaps, analyzing the deformation of the valve flaps and the temporal variations in fluid flow within the pumping mechanism model. The investigation considers different configurations regarding the positions and quantities of the valve flaps, calculating the variations in net volume pumped from left to right over time. This analysis provides valuable insights for microfluidic applications in microscale scenarios. The performance of microfluidic systems is the result of the nonlinear coupling of fluid properties, valve structure, and operating conditions. Future research needs to break through the limitations of a single factor and adopt interdisciplinary methods to achieve system-level optimization, promoting breakthroughs in applications such as organ chips and flexible electronics.

Multiparameter collaborative optimization of microfluidic systems is the core path to break through existing performance bottlenecks and achieve technological breakthroughs. This process requires a deep integration of theoretical modeling, intelligent algorithms, and manufacturing processes to form a closed-loop system from design to validation. Research will be conducted from three dimensions, breakthroughs in key technologies, integration of interdisciplinary tools, and industrial implementation paths, and a quantifiable implementation framework will be provided; multi parameter collaborative optimization of microfluidic systems will break through existing performance bottlenecks and achieve technological leaps. This process requires a deep integration of theoretical modeling, intelligent algorithms, and manufacturing processes, forming a deep development from design.

This study mainly focuses on simulation under specific fluid properties and specific obstacle structure parameters. In practical applications, microfluidic systems may involve fluids of different properties, different obstacle structure designs, and different working conditions. These factors require further research and analysis in the future. In addition, further in-depth research is needed on the formation mechanism, development process, and specific impact on the microparticle performance of complex fluid flow phenomena at the microscale.

## Figures and Tables

**Table 1:** Model configuration data.

Density	Dynamic Viscosity	Horizontal Channel Length	Horizontal Channel Height	Obstacle Width	Obstacle Height	Angle
1000 kg/m^3^	0.001 Pa·s	1000 μm	100 μm	6 μm	56 μm	60°

**Table 2:** Microchannel cross-sectional shape and data (μm).

Channel	Sectional Shape	Width	Length	Height	Equivalent Diameter	Ratio of Length to Equivalent Diameter	Turning Point
1	rectangle	60	1000	100	75	13.3	800
2	rectangle	120	1000	100	109.1	9.2	700
3	Semicircle	200	1000	100	30.54	32.7	1000
4	Semicircle	100	1000	50	15.3	65.4	700
5	triangle	60	1000	100	44.6	22.4	900
6	triangle	120	1000	100	67.9	14.7	900

## Data Availability

The original contributions presented in the study are included in the article, further inquiries can be directed to the corresponding author.
